# Multiple Cranial Nerve Palsy Concomitant with Leptomeningeal Involvement in Multiple Myeloma: A Case Report and Review of Literature

**Published:** 2018-01-01

**Authors:** Neda Pak, Fatemeh Shakki Katouli, Amir Reza Radmard, Mohammad Hassan Shaki Katuli, Mohamad Mehdi Rezwanifar, Negar Sepehri Boroujeni

**Affiliations:** 1 Department of Radiology, Shariati Hospital, Tehran University of Medical Sciences, Tehran, Iran; 2 Department of Cardiology, Tehran Heart Center, Tehran University of Medical Sciences, Tehran, Iran; 3 Department of Pathology, Shariati Hospital, Tehran University of Medical Sciences, Tehran, Iran

**Keywords:** Multiple myeloma, Diagnosis, Neuroimaging

## Abstract

Neurologic symptoms are quite common in multiple myeloma, but direct invasion of central nervous system is extremely rare. Leptomeningeal multiple myeloma, as a rare neurological manifestation of multiple myeloma, presents with impairment of consciousness, cranial nerve palsies and convulsions. Here, we describe a 52-year- old male patient, known case of multiple myeloma, who presented with bilateral visual loss and difficulty in swallowing.

## Introduction

 Multiple myeloma (MM) is a malignant disorder characterized by the proliferation of a single clone of plasma cells in the bone marrow (BM)^1^. The disease typically restricted to the BM and the skeletal system ^2^. 

Neurological complications of MM are not unusual; however, direct involvement of the central nervous system (CNS) is extremely rare^3^. CNS involvement as defined by the detection of malignant plasma cells in the cerebrospinal fluid (CSF) occurs in about 1% of patients and includes localized intra-parenchymal lesion, solitary cerebral plasmacytoma or leptomeningeal multiple myeloma (LMM) ^4^. 

LMM presents with impairment of consciousness, cranial nerve palsies and convulsions. Presence of atypical plasma cells in the CSF is an important finding for the diagnosis of meningeal myeloma^5^. CSF analysis which is abnormal in all patients reveals pleocytosis and elevated protein content in association with positive cytological findings. Specific magnetic resonance imaging (MRI) findings which are suggestive of CNS involvement includes leptomeningeal contrast enhancement and meningeal-based lesions sometimes masquerading as intra-parenchymal lesions^6^.

The reported median interval from initial diagnosis of MM to the CNS involvement is about 11 to 13 months^7^. The prognosis of these patients is very poor, and median survival from the time of diagnosis is 2.0 months^8^^, ^^9^.

**Case presentation**

**Clinical history**

The patient is a 52-years-old male, known case of multiple myeloma from 22 months ago, who presented with bilateral visual loss (more severe in left side) and difficulty in swallowing. He had previously received systemic chemotherapy based on the diagnosis of refractory multiple myeloma.

His first presentation was generalized bone pain. After the confirmation of MM diagnosis, he received VAD regimen (Vincristine, Adriamycin, and Dexamethasone) which resulted in remission (normal serum protein electrophoresis and less than 5% plasma cell in bone marrow aspiration). After remission, at the time he was candidate for bone marrow transplantation, the myeloma relapsed with presentation of multiple cutaneous nodules. This time the patient underwent VTD regimen chemotherapy (Velcade, Thalidomide and Dexamethasone) again. About one month later, he admitted to emergency department with neurologic complaints.

Abnormal neurologic examination at the time of admission included left eye blindness with optic disc atrophy, pupil mydriasis and non-reactivity, right eye blurred vision with pupil mydriasis and reactivity, a decrease in gag reflex, a decrease of muscle forces in upper and lower limbs (4+ and 4-, respectively), the absence of deep tendon reflex, waddling gait and impaired tandem gait. Mental status was normal.

**Imaging**

**Figure 1:**
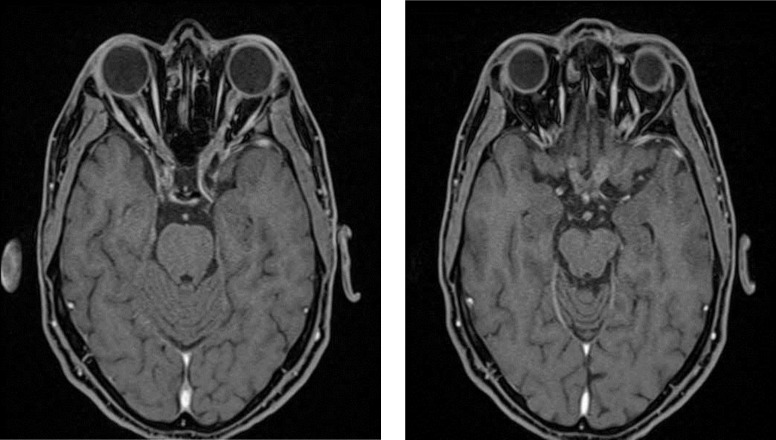
In T1- weighted post-contrast images, there is thick and nodular contrast enhancement along the optic nerves (left) and the optic chiasm (right)

**Figure 2:**
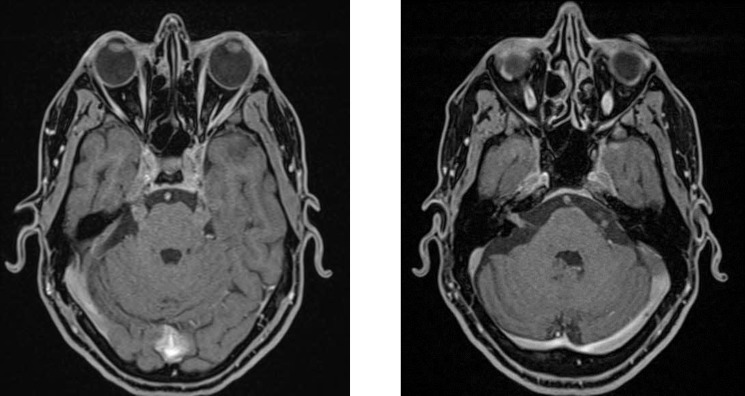
In T1 -weighted post- contrast images, there is diffuse bilateral nodular contrast enhancement along the 5th (left), 7th and 8th cranial nerves (right)

Brain and orbital MRI with administration of contrast media showed diffuse nodular enhancement and thickening along the most of cranial nerves, including optic nerves and optic chiasm (Figure 1), 5th, 7th, 8th, 9th nerves (Figure 2), Meckel’s caves and cavernous sinuses. Moreover, a 9*10mm enhancing extra-axial meningeal-based nodule adjacent to right paracentral gyrus and some enhancing osseous lesions in clivus and right occipital condyle were detected due to MM.

**Laboratory findings**

On the first admission, the supportive lab finding for multiple myeloma was M-spike in late gamma region in protein electrophoresis. After first relapse, an abnormal band in the late-gamma region was detected.

Laboratory data at the first day of admission were as follows: 

Blood chemistry as: BUN=19, Cr=0.7, Na=142, k=4.8, Ca=8.9, AST=20, ALT=15, ALP=81

-   Peripheral blood as: WBC count=6700 , Hemoglobin= 14.2 , platelets= 229000

-   CSF analysis as: protein=397 , glucose=58 , RBC=52 ,WBC=425 (many plasma cells) , LDH=57

-   Negative CSF smear and culture

**Figure 3:**
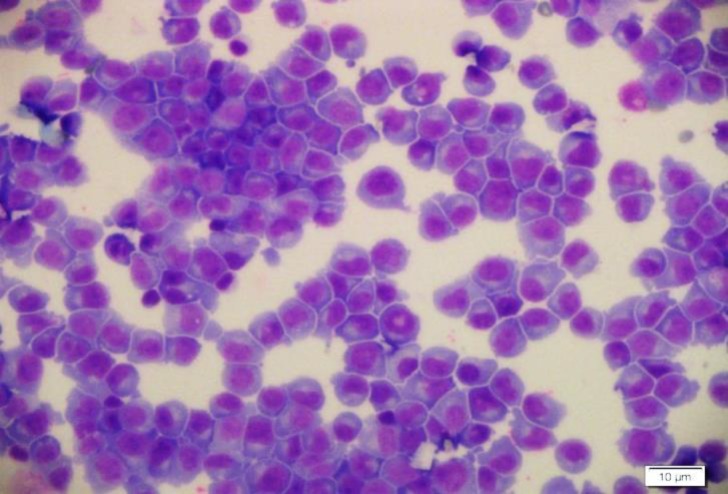
In microscopic exam, sheets of plasma cells were identified.

**Figure 4:**
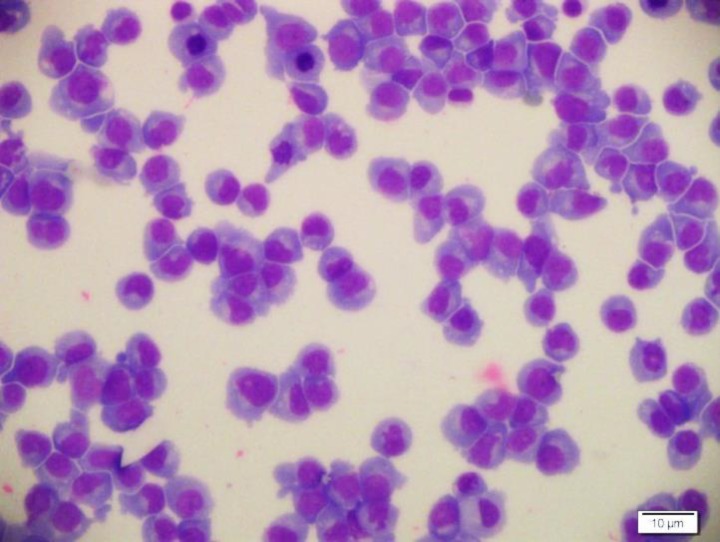
Aggregates of plasmacytoid cells. Some mitotic figures are present.

The CSF specimen was semi-clear in the gross examination, so the sample was centrifuged, and then slides were prepared and stained by Wright method. In microscopic exam sheets of plasma cells were identified (Figures 3, 4).

Considering the underlying disease, plasma cell infiltration of cranial nerves was considered as the top differential diagnosis and confirmed by CSF analysis. The presence of monoclonal plasma cells in CSF analysis proved CNS involvement. 

**Therapeutic approach**

After diagnosis of LMM, treatment changed to intrathecal chemotherapy. After one course, because of severe spasticity, intrathecal chemotherapy was stopped, and the therapeutic plan changed to radiotherapy. 

**Literature review**

Multiple myeloma represents a heterogeneous collection of several different cytogenetically distinct plasma cell malignancies. Neurologic symptoms are quite common in MM including spinal cord compression by a soft-tissue plasmacytoma or bone fragments of a fractured vertebral body, metabolic encephalopathy secondary to hypercalcemia or uremia, peripheral neuropathies in the context of amyloidosis or treatment toxicities and hyper viscosity symptoms. Usually, positive CSF analysis associated with plasma cells is considered as a confirmatory evidence of leptomeningeal involvement. In our review, all cases had a positive CSF analysis for malignant cells. Several mechanisms have been proposed for LMM including direct invasion from contiguous involved bone (by a lytic lesion erodes the skull and dura matter) or hematogenous spread as seen in plasma cell leukemia ^10^^-^^14^. This review of literature yielded 9 articles including 11 different patients reported of MM with leptomeningeal involvement and cranial nerve palsies. Six cases were male and 5 were female. The average age at the time of diagnosis was 59 years. There were 2 cases of IgA myeloma subtype who showed the highest CSF plasma cell count (548 * 10^6^ / liter), either the highest CSF protein level (185 g/dl) in comparison with other subtypes. All patients had received intrathecal chemotherapy. Unfortunately, most of the patients had a poor prognosis after confirmation of leptomeningeal involvement by CSF analysis. The mean overall survival after CNS involvement was 3.5 months, (rang 1 - 9 months). Similar results were reported by Nieuwenhuizen et al^9^. They summarized the clinical and laboratory characteristics and treatment modalities of 109 patients with LMM, and demonstrated that the overall median survival from the time of diagnosis to death was 2.0 months (range 0.1-25 months). Patients who were treated with cranial irradiation (CI) had a significantly (P = 0.004) longer survival compared with patients without CI. In this review, 9 patients had only one cranial nerve palsy (most commonly second cranial nerve), while 2 patients had multiple cranial nerve palsies and concurrent involvement of 3rd, 5th and 7th nerves were reported in both of them. Although direct involvement of cranial nerves has been reported in the course of refractory MM and it is very rare at initial presentation, such combinations of cranial nerve involvement (2nd, 5th, 7th, 8th, 9th, and 10th) and MRI features have made this case interesting. MRI is a useful tool in assessment of such patients, although may be negative in about 10% of patients and the diagnosis should be confirmed by CSF analysis in all patients. The usual differential diagnosis of LMM in imaging studies includes metastasis, lymphoma, plasmacytoma, meningioma, dural granulocytic sarcoma, infectious meningitis, and leptomeningeal carcinomatosis^15^. Unfortunately, no guideline has been established for treatment of LMM. Many novel strategies are being employed to increase efficacy of treatment in MM. Some recent combination therapies are IMiD and PI–based combination regimens, HDACI-based combination regimens and antibody-based combination regimens ^16^. But, there is also concern about adverse effects of these new programs like extra medullary plasmacytoma and CNS myelomatosis^9^^,^^17^^,^^18^. Many other engineered immune response based agents against MM are in preclinical models that may soon make their way to the clinic^19^. Finally, we suggest a prospective, multicenter, randomized trial for evaluation of several treatment modalities for this rare but tragic disease entity.

**Table 1:** Results of review the literature

Report	Case	Age/Sex	Myeloma type	Cranial nerve palsies	Interval from Diagnosis to Developm ent of Meningeal disease (month)	CSF		Treatment			Response	Autopsy	Survival from diagnosis of mening eal disease (month)
						Plasma cells (* 106 / iter)	Protein (g /dl)	Cranial irradiation	Intrathecal chemotherapy	Corticosteroids			
Woodruff, R K20 (1982)	1	69 / M	IgG	3,5,7,9,10	17	47	NA	**+**	**+**	**+**	**-**	**+**	NA
Leifer, D21 (1992)	1	65 / F	Kapp a Light chain	NA	7	186	0.93	**-**	**+**	**+**	**-**	**-**	**3**
	2	69 / F	IgG Lamb da	3,5,7,8	24	90	5.2	**-**	**+**	**-**	**-**	NA	**1.5**
	3	54 / M	IgG Lamb da	NA	5	566	9.25	**+**	**+**	**+**	**-**	**+**	**5**
Pizzuti, P22 (1997)	1	54 / M	Kapp a	NA	0	9	1.45	**-**	**+**	**-**	**-**	NA	**1.5**
Roddie, P23 (2000)	1	55 / F	Kapp a	5	15	0	1.13	**-**	**+**	**-**	**+**	**-**	**-**
Yeung, SoniaN24 (2008)	1	69 / M	IgG	2	6 yrs	NA	NA	**+**	**+**	**+**	**-**	**+**	**1**
Riley, Jenny M25 (2011)	1	58 / F	IgAk	2	14	NA	NA	**+**	**+**	**+**	**-**	NA	NA
Grisold, A26 (2014)	1	66 / M	Lamb da Light chain	6	20	275	83	**-**	**+**	**-**	**-**	**-**	**9**

## CONCLUSION

 MRI is a useful tool in assessment of LMM, although may be negative in about 10% of patients and the diagnosis should be confirmed by CSF analysis. Presence of atypical plasma cells in the CSF is an important finding for the diagnosis of LMM. To confirm the diagnosis and evaluate the extent of disease, using MRI and lab tests are recommended.
